# Insights into the molecular and genetic role of obesity in breast cancer pathogenesis

**DOI:** 10.1080/15384047.2025.2501345

**Published:** 2025-05-12

**Authors:** Sandeep Mallya, Varsha Gangadhar, Sophia Evangeline Aldrin, Meghana Acharya, Shama Prasada Kabekkodu, Kiran Kumar Kolathur, Sanjiban Chakrabarty

**Affiliations:** a Department of Bioinformatics, Manipal School of Life Sciences, Manipal Academy of Higher Education, Manipal, India; b Department of Cell and Molecular Biology, Manipal School of Life Sciences, Manipal Academy of Higher Education, Manipal, India; c Department of Pharmaceutical Biotechnology, Manipal College of Pharmaceutical Sciences, Manipal Academy of Higher Education, Manipal, India; d Department of Public Health and Genomics, Manipal School of Life Sciences, Manipal Academy of Higher Education, Manipal, India

**Keywords:** Obesity, breast cancer, molecular subtypes of cancer, epidemic, hormonal imbalance, breast cancer pathogenesis, molecular and genetic role of obesity

## Abstract

The epidemic of obesity is a growing concern and is one of the major risk factors for several chronic diseases, including several types of cancers. The correlation of breast cancer with obesity has been extensively studied and involves an interplay of hormonal, metabolic, and genetic factors explored in this review. Inflammation and hormone dysregulation play an important role in promoting a protumorigenic environment through adipose tissue, which is involved in energy storage and functions as an endocrine organ. As a result, various cytokines, primarily proinflammatory in nature, are released, resulting in low-grade inflammation promoting tumor growth. Additionally, obese conditions also induce imbalances in hormones, particularly estrogen and insulin, both of which drive carcinogenesis. Genetic components such as single nucleotide polymorphisms also play critical roles in modulating the correlation between obesity and breast cancer. This review provides a comprehensive overview of various mechanisms underlying obesity and breast cancer incidence and progression.

## Introduction

An imbalance in the energy intake and expenditure leads to excessive accumulation of body fat, causing obesity.^1^ Obesity, occurring in approximately 15% of the world’s adult population, is associated with several comorbidities including diabetes, hypertension, cardiovascular diseases and certain types of cancers.^2,3^ The absence of or reduction of body fat has also been associated with a reduction in the risk of a number of cancer types, including breast (postmenopausal), bowel, esophagus, liver, ovary, endometrium, gastric cardia, gallbladder, pancreas, kidneys, meningioma, multiple myeloma, and thyroid cancers.^3^ Specifically, several studies on breast cancer have revealed a link between obesity and a higher risk of recurrence in postmenopausal women.^4^

Cancer poses a significant global burden, accounting for close to ten million deaths in the year 2022. The most common cancers, lung, breast and cervical, contribute to greater than 50% of deaths worldwide. The International Agency for Research on Cancer (IARC) in their report GLOBOCAN estimated that in 2022, in excess of 2.3 million women were diagnosed with breast cancer accounting for 665,684 deaths making it the most common cancer among women, followed by cervical cancer.^5^ Several factors influence the incidence of breast cancer, including genetic, environmental and lifestyle factors such as alcohol consumption, physical activity status, obesity and overweight. Specifically, 21% of breast cancer risk is influenced by overweight and obesity.^6^

Adipocytes constitute the integral component of the interstitial tissue of the mammary gland, of which the percentage of adipose tissue to total breast volume varies from 7% to 56%, and the adipose tissue weight accounts for approximately 3.7% to 37% of the total weight.^7^ These adipocytes secrete adipokines, which play crucial roles in the development of the breast through differentiation of the epithelium.^8^ Obesity leads to the expansion of adipose tissue due to increased energy intake. This further expansion can occur through hypertrophy or hyperplasia. Hypertrophy is the expansion of tissue, leading to a proinflammatory environment with increased levels of cytokine production and fatty acid release, which is further associated with the dysfunction of adipose tissue, causing tumorigenesis.^9^ Additionally, the adipose tissue surrounding cancer cells plays a coordinated and complex role in tumor progression through angiogenesis, enhancement of cell migration, apoptosis and genomic instability, and this communication occurs through the release of various cytokines and adipokines.^10^ They play important roles in maintaining the immune landscape of the tumor microenvironment (TME) and remodeling of the extracellular matrix (ECM), facilitating metastasis.^11^

## Role of obesity in patients with breast cancer according to molecular subtype

Breast cancer is characterized by heterogeneity within the tumor cell population, with diverse genetic, epigenetic, and transcriptomic profiles. As a result of heterogeneity, cancer is classified into various molecular subtypes based primarily on their gene expression patterns of hormone receptors and growth factors.^12,13^ For the molecular classification of breast cancer into luminal A, luminal B, HER2-enriched, and triple-negative subtypes, three receptors – estrogen receptor (ER), progesterone receptor (PR), and human growth factor receptor 2 (HER2) are used.^13^

Luminal breast cancers are characterized by ER positivity and 70% of all breast cancer cases are of the luminal A subtype.^14^ In these tumors, obesity is associated with reduced overall survival (OS) and relapse-free survival (RFS).^15^ Premenopausal obese women have 30% lower risk of developing luminal A breast cancer,^16^ whereas postmenopausal women with a higher body mass index (BMI) have a higher risk of developing luminal B tumors. An inverse relationship between obesity and breast cancer is observed in premenopausal women, where obesity is associated with a 26% reduction in the risk of developing ER+ breast cancer.^16,17^ Additionally, the proportion of obese patients with breast cancer with PR+ tumors was also found to be higher than that of nonobese patients^15^

Cancers, including breast cancer, are influenced by the HER family of receptors, including HER1, HER2, HER3, and HER4. About 15%-20% of breast cancer cases result in the overexpression or gene amplification of HER2.^18,19^ While the impact of obesity is well established with respect to breast cancer progression, its association with HER2-positive breast cancer risk remains unclear.^20^ However, a meta-analysis suggested that underweight premenopausal women may have an increased risk of developing HER2-positive breast cancer.^16^ Additionally, higher BMI is associated with lower survival rates in early HER2-positive breast cancer patients; conversely, in advanced HER2-positive breast cancer patients, higher BMI may be correlated with better survival.^21^

Triple-negative breast cancer (TNBC) is a subtype of breast cancer characterized by a lack of ER, PR and HER2 expression. This accounts for TNBC being unresponsive to hormone therapy and HER2-targeted therapies.^22^ Approximately 75% of the breast cancers associated with BRCA1 mutations are TNBCs.^23^ Obesity is more frequently associated with TNBC than with other breast cancer subtypes. A study conducted in West Virginia reported that approximately 49.6% of TNBC patients were obese at diagnosis and the Carolina Breast Cancer Study revealed a greater risk of TNBC development in both pre- and postmenopausal women with a greater waist-to-hip ratio (WHR); however, BMI was not a significant factor.^24^ Similarly, in a study conducted with African American women, a higher WHR was linked to a high risk of TNBC, whereas BMI alone was not associated with TNBC risk.^25^ Additionally, obesity-mediated inflammatory conditions lead to increased levels of the cytokine IL-6, which is associated with the progression of tumor growth through the activation of the IL-6/STAT3/PTX3 pathway.^26^ However, the impact of obesity on TNBC risk and prognosis may vary and needs to be explored further.^27^

## Molecular links between breast cancer and obesity

**Figure 1.**
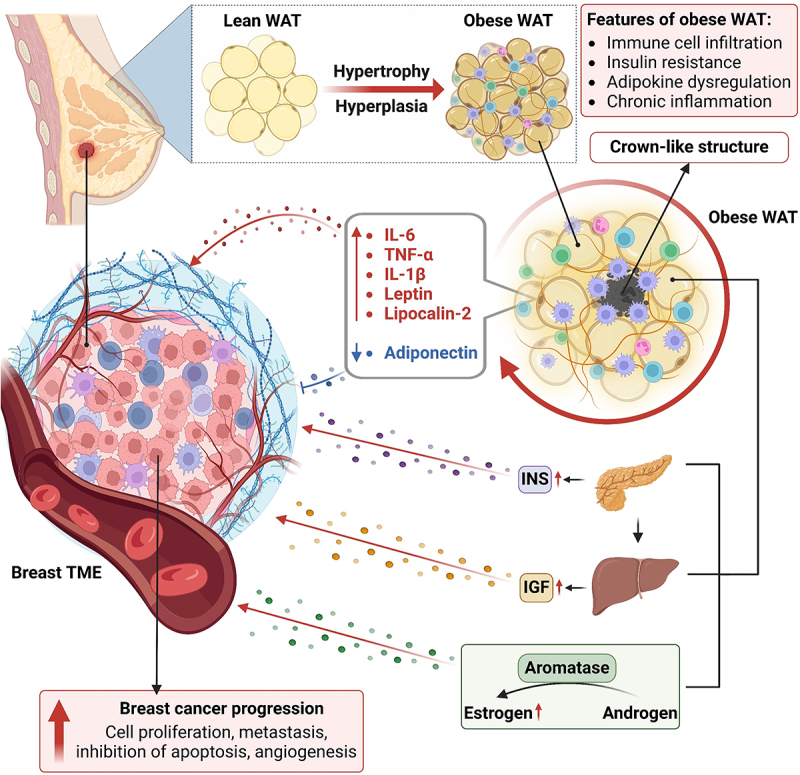
Role of obesity in breast cancer. Obesity is linked with hypertrophy and hyperplasia of adipose tissue. Obese WAT promotes the secretion of various inflammatory cytokines and sex hormones and adipokine dysregulation, increases insulin and IGF levels, and triggers chronic inflammation, all of which contribute to the creation of a favourable tumor microenvironment. Obese WAT is infiltrated by immune cells, with macrophages surrounding dying adipocytes, forming crown-like structures (CLSs), and further contributing to inflammation. The overexpression of aromatase in adipose tissue increases estrogen production. Obesity-related insulin resistance and hyperinsulinemia drive breast cancer progression. Figure created with BioRender.com.

The relationship between breast cancer and obesity is multifaceted and influenced by factors such as menopausal status and tumor subtype.^28^ Breast cancer is particularly influenced by adipose tissue due to the histological structure of the breast tissue, with the local and systematic obese environment facilitating oncogenesis.^29,30^ Approximately five hundred biologically active factors are produced by adipose tissue, of which a few key factors, including interleukin-6 (IL-6), visfatin, resistin, and tumor necrosis factor (TNF-a), alter the levels of anti-inflammatory and proinflammatory factors.^31^ These factors are further involved in the regulation of various processes, such as insulin sensitivity and secretion, homeostasis, the immune response, the accumulation of immune cells, inflammation, and vascular growth and function.^31,32^ Under obese conditions, the expansion of white adipose tissue (WAT) due to the accumulation of fat in adipose depots alters the physiology, metabolism, epigenetics and transcriptomics of breast adipose tissue. Dysfunction of this tissue is further linked to various features associated with carcinogenesis. Broadly it can be classified into three major impacts, which have been reported as causes of breast cancer, including chronic inflammatory conditions developed due to the accumulation of macrophages and excess cytokines, excess accumulation of estrogen due to increased production of aromatase by adipose tissues and increased levels of insulin due to insulin resistance and hyperinsulinemia (Figure 1).^3^

## Role of adipocytokines in obesity and breast cancer

Excess accumulation of fat under obese conditions leads to the incidence of chronic inflammatory conditions, which further results in an inflammatory TME.^33^ Complex cross-talk among the different components within the TME promotes tumorigenesis. The cells include cellular and extracellular factors. The cellular component consists of stromal cells, epithelial cells, fibroblasts, pericytes, mesenchymal stem cells and diverse immune cells.^34,35^ Macrophages, which constitute a part of the TME, usually constitute approximately 10% of adipose tissue. This level is increased to approximately 40% in cases of obesity.^33^ This accumulation of macrophages further leads to increased uptake of glucose by adipocytes, which results in further inflammation and adipocyte dysfunction.^36^

Dysfunction of adipose tissue leads to adipose tissue inflammation (ATI), which alters the adipocytokine balance, promoting the production of proinflammatory cytokines such as interleukin-6 (IL-6) and tumor necrosis factor-a (TNF-α) and the activation of the nuclear factor (NF)-κB pathway and leptin, with each of these factors contributing to carcinogenesis^37^ as described in Table 1.

**Table 1.** Impact of obesity on the levels of adipokines and cytokines and their implications in breast cancer prognosis.

Cytokine/Adipokine	Observation	Impact
IL-6	Upregulated	Proinflammatory Activation of JAK1/STAT3 pathway Activation of oncogenes PD-L1, IDO1 Angiogenesis through upregulation of VEGF
TNF-α	Upregulated	Proinflammatory Hyperinsulinemia and upregulation of aromatase thus increase in estrogen levels Activation of cell proliferation pathways - MAPK, AP-1, JNK, NF-κB
IL-1β	Upregulated	Marker for metastasis Promotes epithelial to mesenchymal transition (EMT) through activation of MMP-9 Activation of ERK1/2 pathway and EGF signaling Angiogenesis through upregulation of VEGF
Leptin	Upregulated	Proinflammatory Activation of STAT3 Promotes expression of TNFα and IL-6 Angiogenesis through upregulation of VEGF Promotes expression of hTERT
Adiponectin	Downregulated	Anti-inflammatory Promotes invasion of tumors

## Sex hormone dysregulation and its effects on obesity and breast cancer

Approximately 70% of breast tumors express progesterone and estrogen receptors, which contribute to hormone-dependent breast cancer. Increased exposure to these hormones leads to hormone-dependent breast carcinogenesis.^28^ Estrogen is linked to the initiation and progression of breast tumors, and fluctuating estrogen levels throughout a female’s life render the breast tissue vulnerable to inflammation.^38,39^

**Figure 2.**
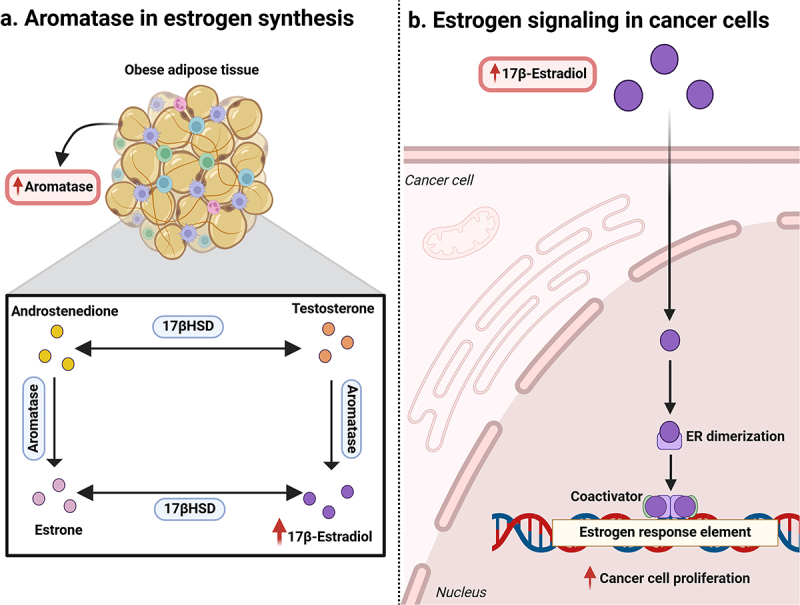
Estrogen production in adipose tissue and estrogen signalling. (a) Role of aromatase in estrogen synthesis: In obese adipose tissue, increased aromatase activity, which is involved in the conversion of androgens (androstenedione and testosterone) into estrogen (estrone and 17β-estradiol), is observed. Estrone is further interconverted to 17β-estradiol, forming a potent estrogen. (b) Estrogen signalling in cancer cells: elevated levels of 17β-estradiol activate estrogen receptors (ERs) and lead to ER dimerization, which further leads to the activation of estrogen response element (ERE) controlled mechanisms associated with cell growth and proliferation. Figure created with BioRender.com.

The ovaries are the primary source of estrogen production in premenopausal women. This process is further regulated by follicle-stimulating hormone (FSH), which is secreted by the pituitary gland and is involved in increased production of estrogen in ovaries.^40^ However, in postmenopausal women, the ovaries produce negligible amounts of estrogen. Instead, peripheral adipose tissue becomes the primary site of estrogen biosynthesis (Figure 2), where the most prevalent steroids are circulating dehydroepiandrosterone (DHEA) and androstenedione.^41^ This forms the precursor for estradiol production. DHEA, or its sulfated form, DHEA-S, is converted to androstenedione by the enzyme 3-β-hydroxysteroid dehydrogenase (3βHSD). Androstenedione can further be converted to testosterone via the 17βHSD enzyme. However, both forms of androgens are converted to estrogen through the activity of aromatase.^42^ The enzyme aromatase, which is a cytochrome P450 enzyme encoded by the CYP19A1 gene, plays a key role in the biosynthesis of estrogen. It converts excess androgen into estrogen via aromatization.^40^ Under obese conditions, WAT elevates aromatase expression and consequently estrogen production. Additionally, obese WAT enhances estrogen availability by reducing the levels of sex-hormone-binding globulin (SHBG).^39^

Various studies have concluded that estrogen promotes tumor development in breast tissue. Estrogen enhances signaling pathways that promote cell proliferation and inhibit apoptotic activity.^38^ The estrogen receptor (ER) is stimulated by estrogen and plays a critical role in breast tumorigenesis.^43,44^ The nuclear estrogen receptor (nER) and the G protein-coupled estrogen receptor (GPER) are both members of the ER family. The ESR1 and ESR2 genes, respectively, encode the estrogen receptor α (ERα) and estrogen receptor β (ERβ) ER isoforms.^45^ These isoforms of ERα receptors (ERα66 and ERα36) are implicated in BC progression and treatment resistance. Approximately 70% of BCs express ERα66, which promotes resistance to antiestrogen treatments.^46^ The expression levels of ERα36 in ER-positive and ER-negative tumors are associated with histological grade and metastasis.^47^ Research has shown that in the TNBC subtype, the expression level of ERα36 is correlated with tumor growth, progression and metastasis.^46^

## Role of insulin and IGF-1

Increased circulating insulin levels and the overexpression of IGFs are often consequences of obesity.^30,48^ Excess visceral adipose tissue (VAT) leads to insulin resistance and hyperinsulinaemia.^43^ Hyperinsulinemia is correlated with an increased risk of developing BC and poor prognosis in obese BC patients.^28^ Insulin binds to insulin receptors (IRs) expressed on the surface of tumor cells. High circulating insulin levels in obesity-related tumors disrupt the insulin signaling pathway and alter IR expression levels.^28^ Obese women with high insulin levels are at greater risk of developing BC. The overexpression of IR in breast tumors indicates that cancer cells are sensitive to elevated insulin levels and is associated with poor patient prognosis.^30,44,48^

**Figure 3.**
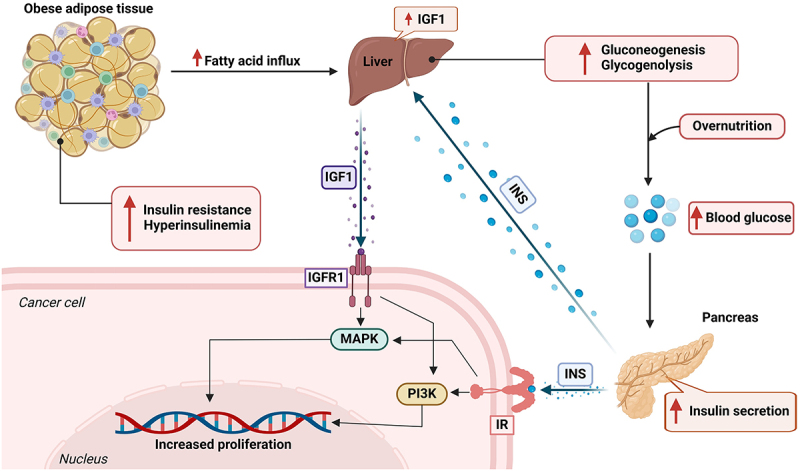
Insulin resistance and hyperinsulinemia are observed under obese conditions and are accompanied by increased influx of fatty acids into the liver. The liver further produces high levels of IGF1, which acts through its receptor IGFR1 on cancer cells. This activation further triggers the MAPK and PI3K pathways, which are involved in proliferation. Hyperinsulinemia also leads to increased blood glucose levels through gluconeogenesis and glycogenolysis in the liver. Increased glucose levels further trigger insulin secretion in the pancreas, which further acts on IR in cancer cells, enhancing cell proliferation pathways. Figure created with BioRender.com.

The insulin growth factors IGF1 and IGF2 (encoded by the IGF1 and IGF2 genes, respectively) are involved primarily in activating the IGF1 receptor (IGF1R).^43,45^ IGF1 and IGF2 are peptide hormones that are functionally similar to insulin and are secreted in response to growth hormones. These hormones aid in lowering blood glucose levels and enhancing cell proliferation. Breast tumors express IGFR six fold higher than normal breast tissue. Studies have found that IGF2 levels are higher than normal in morbidly obese patients.^44^ Besides, insulin can also bind to IGF1R and activate mitogenic pathways (PI3K – AKT – mTOR pathway and MAPK/ERK pathway) leading to cellular growth and proliferation. Research has shown that insulin and IGF also supports the development and functioning of cancer stem cells (CSCs), which contributes to tumor growth and metastasis.^48^ Several preclinical studies have shown that PI3K – AKT – mTOR pathway contributes to endocrine therapy resistance in breast cancer (Figure 3).

Tumor cells adopt the Warburg effect to meet the increasing demands for energy in an oxygen-deprived tumor microenvironment. In the Warburg effect, tumor cells transition their primary energy source from oxidative phosphorylation to glycolysis.^46^ This metabolic shift also facilitates tumor progression by providing building blocks such as ATP and nucleotides for rapid proliferation. In tumor cells, glucose-6-phosphate, an intermediate in the glycolysis pathway, enters the pentose phosphate pathway and acts as a precursor for DNA synthesis and NADPH production. Aberrant mTOR also increases glucose uptake via GLUT1, a glucose transporter. The overexpression of GLUT1 is associated with poor prognosis.^46,48^

## Genetic connections linking obesity and breast cancer

Breast cancer is heterogeneous in nature, and along with molecular dysfunction, it can also be associated with genetic variations. The association with genetic factors is usually observed in patients with a family history due to the inheritance of autosomal dominant cancer susceptibility.^47^ The important inherited mutations include those in the BRCA1 and BRCA2, TP53, phosphatase and tensin homologue (PTEN), serine-threonine kinase-11 (STK11/LKB1), and cadherin 1-type 1 (CDH1) genes.^49,50^ Approximately 25% of breast cancers are considered hereditary and are caused by mutations in one of the aforementioned genes. These mutations account for nearly 80% of the genetic risk associated with hereditary breast cancer.^50^ However, the associations of these genes with the onset of obesity and its correlation with the incidence of breast cancer are still being studied and are not well understood. However, certain obesity-associated genes, such as proliferator-activated receptor-γ (PPARG), lipoprotein lipase (LPL), leptin receptor (LEPR), paraoxonase (PON1 and PON2) and tumor necrosis factor-α (TNF-α), are also being further investigated for possible associations as risk factors for breast cancer development.^51–53^ Single nucleotide polymorphisms (SNPs) within these genes can increase breast cancer risk by altering the expression of DNA repair genes, changes in hormone receptor expression and pathways involved in tumor growth and progression. The SNPs observed in these genes are summarized in Table 2. However, no statistically significant correlation with breast cancer risk has been consistently reported across studies. Additionally, obesity can also induce epigenetic variations, which play a significant role in carcinogenesis either by activating oncogenes or through the loss of tumor suppressor genes.^47^

**Table 2.** SNPs and mutations of genes and their correlation with breast cancer risk.

Gene	SNP	Association with breast cancer	Reference
FTO	rs9930506	Decreased risk	^54^
	rs9939609	Increased risk	^55^
	rs1477196	Increased risk	^55^
	rs7206790	Increased risk	^55^
	rs8047395	Increased risk	^55^
MC4R	rs17782313	Increased risk	^56^
BRCA1/BRCA2	Mutation	Increased risk	^57,58^
LEP	rs1137101	Increased risk (with G allele)	^59^
		Decreased risk (with A and GA+AA)	^60^
PON	rs662	Increased risk	^52^
	rs854560	Increased risk	^52^

Several genome-wide association studies (GWASs) have recently reported the associations of fat mass and the obesity-associated (FTO) gene with obesity, breast cancer and diabetes.^61,62^ FTO has been associated with the DNA damage response, DNA repair and inflammatory mechanisms.^2^ It encodes a protein that functions as a nucleic acid demethylase, targeting N6-methyladenosine (m6A) modifications on RNA. m6A is a cotranscriptional modification in eukaryotic RNA, that plays a role in regulating the stability, splicing and transport of RNA. An imbalance in m6A modification is associated with several physiological and pathological processes, including obesity and cancer.^63^ FTO is highly expressed in the hypothalamus, brain and pancreatic beta cells; thus, its role in the regulation of insulin secretion has been suggested.^64^ The SNP rs9939609 (T/A substitution) found in intron 1 of the FTO gene is correlated with a preference for high dietary fat intake in patients with obesity due to its altered expression in the hypothalamus.^65,66^ Although FTO can act as an oncogene upon overexpression, a recent study revealed no association between polymorphisms within the FTO gene and breast cancer risk but was able to conclude that the AG phenotype of the rs9930506 SNP was associated with a reduced incidence of breast cancer, establishing a protective role.^54^ In contrast, studies have also demonstrated the associations of several SNPs, including rs9939609, rs1477196, rs7206790, and rs8047395, with the incidence of breast cancer.^55^

Melanocortin 4 receptor (MC4R) is another obesity-associated gene that is located on chromosome 18q22 and consists of a single exon. MC4R further codes for a transmembrane G protein-coupled receptor containing seven domains that is highly expressed in adipose tissue, muscle and the brain. Like FTO, MC4R is also associated with food intake requirements and energy balance within the body.^56^ In 2008, a GWAS reported a strong association between the SNP rs17782313 and obesity risk in European populations.^56,67^ A recent meta-analysis investigated the association of this polymorphism with cancer incidence and revealed that it was moderately associated without BMI adjustment. However, there was no association when adjusted for BMI, implying that cancer risk may be mediated through obesity. However, no correlation was found with the incidence of breast cancer via organ-specific analysis.^56^

BRCA1 and BRCA2 are proteins that form essential components of the homologous recombination pathway involved in the DNA damage response, enabling DNA double-strand break repair. Germline mutations within genes 1 and 2 result in the accumulation of double-strand breaks, increasing susceptibility to tumorigenesis due to genomic instability.^68,69^ Approximately 10% of breast cancer cases are hereditary in nature due to germline mutations in BRCA genes, which are associated with early-onset breast cancer and an increased risk of other cancer types.^69^ BRCA mutation carriers have a 70% risk of developing breast cancer by the age of 80 years.^68^ Additionally, screening for this germline mutation plays a crucial role in treatment selection, as individuals with BRCA germline mutations have increased sensitivity to PARP inhibitors and platinum-based chemotherapies and decreased sensitivity to CDK4/6 inhibitors.^68,70^

Obesity is also associated with increased DNA damage due to the generation of reactive oxygen species (ROS). Moreover, the DNA damage repair capacity decreases with increasing BMI, thus acting as a cancer risk factor.^132^ Obesity or higher BMI is also associated with the accumulation of DNA double-stranded breaks in normal breast epithelial cells in BRCA mutation carriers, thus increasing the risk of carcinogenesis and indicating the effect of obesity on cancer development.^57,58^

Leptin (LEP), which is an adipokine, plays a crucial role in energy expenditure and food intake, and its plasma level reflects body fat mass. Leptin regulates energy homeostasis through the leptin receptor (LEPR), and the overexpression of LEP and LEPR links obesity with breast cancer.^133^ A study conducted with 320 female subjects from Jordan investigated the impact of the SNP rs1137101 within the LEPR.^59^ rs1137101 is associated with an A to G substitution in LEPR, which alters leptin binding, thus leading to increased serum leptin. The study revealed statistically significant differences in the presence of the G allele between breast cancer patients and controls (approximately 60.6%). Conversely, the A allele and GA+AA phenotype were found to be correlated with decreased breast cancer risk in a study conducted among the Chinese population.^60^ This polymorphism is correlated with obesity, and individuals carrying the G allele show significant weight gain in both adults and children and can act as a predictor of rapid weight gain in children.^134^ Another study associated the presence of the G allele with an increased risk of adiposity in the Sri Lankan population.^135^

Three proteins, PON1, PON2 and PON3 of the paraoxonase (PON) family are characterized by multiple functions, including protection against oxidative stress and ER stress, lipid peroxidation, and detoxification, along with anti-inflammatory properties and apoptotic-related modifications. The impact of obesity on the activity of PON1 follows an inverse relationship. Increased levels of leptin under obese conditions lead to decreased PON1 activity. Leptin, which is secreted at elevated levels due to adipocyte dysfunction, leads to a decrease in high-density lipoprotein (HDL) and triglyceride levels, which in turn reduces the fractions of HDL, thus leading to the underexpression of PON1.^136^ A genetic alteration in the genes involved in oxidative stress pathways can increase the risk of breast cancer due to the role that oxidative stress plays in the development of breast cancer.

Several SNPs within the PON1 gene have been identified, of which rs662 and rs854560 have been frequently studied.^52,137,138^ A meta-analysis revealed that both of these SNPs were associated with an increased risk of breast cancer.^52^ Compared with the L isoform, the L55M alteration leads to decreased levels of PON1 mRNAs, and a case-control study among the Iranian population suggested that the presence of this alteration is a risk factor for breast cancer.^137^ Another meta-analysis also revealed a significant association between SNPs and breast cancer risk.^138^ However, the interplay between genetic variations, such as those in the PON1 gene, and obesity suggests a complex relationship that warrants further investigation.

It is also seen that gene expression changes also play an important role in obesity and breast cancer datasets. In Table 3, it is seen that there are several distinct yet overlapping patterns of gene expression between the two conditions. Understanding these interactions could lead to more targeted approaches in predicting and managing breast cancer risk in obese individuals. Future studies focusing on larger, more diverse populations are essential to unravel the nuances of these associations.

**Table 3.** List of commonly regulated genes associated with obesity and breast cancer and their expression patterns under each condition.

Gene symbol	Gene name	Breast cancer	Obesity	Ref
CYP2E1	Cytochrome P450 isoform 2E1	Overexpressed	Underexpressed	71,72
ADIPOQ	Adiponectin	Underexpressed	Underexpressed	73,74
ADIPOR1, ADIPOR2	Adipokine receptors	Underexpressed	Underexpressed	73,75
ADRB2	Beta-2 adrenergic receptor	Overexpressed	Underexpressed	75,76
AKT1	Protein kinase B alpha	Overexpressed	Overexpressed	72,77
BCL2	B-cell lymphoma 2	Overexpressed	Underexpressed	78,79
DIO3	Type 3 deiodinase	Underexpressed	Overexpressed	80,81
E2F1	E2F transcription factor 1	Underexpressed	Overexpressed	82,83
EGFR	Epidermal growth factor receptor	Overexpressed	Overexpressed	84,85
EPHX1	Xenobiotic biotransformation (microsomal epoxide hydrolase)	Overexpressed	Overexpressed	72,86
ERBB2	ErbB2 receptor tyrosine kinase 2	Overexpressed	Overexpresed	87,88
ESR1/ER	Estrogen receptor α	Overexpressed	Mixed: Overexpressed/Underexpressed	89–92
FOXO3A	Forkhead transcription factor subfamily 3	Overexpressed	Underexpressed	72,93
FTO	Fat mass and obesity associated gene	Overexpressed	Overexpressed	94,95
GATA3	GATA binding protein 3	Underexpressed	Overexpressed	96,97
GSTM1	Glutathione S-transferase isoform mu 1	Overexpressed	Underexpressed	72,98
GSTP1	Glutathione S-transferase Pi 1	Overexpressed	Underexpressed	72,99
IGF1	Insulin-like growth factor-I	Overexpressed	Overexpressed	100,101
IL-6	Interleukin 6	Overexpressed	Overexpressed	102,103
IRS1	Insulin receptor	Overexpressed	Underexpressed	72,104
LEP	Leptin	Overexpressed	Overexpressed	105,106
LEPR	Leptin receptors	Overexpressed	Overexpressed	105,107
MAP2K4	Mitogen-activated protein kinase kinase 4	Overexpressed	Overexpressed	108,109
MAP3K8	Serine threonine mitogen activated protein kinase kinase kinase 8	Overexpressed	Overexpressed	93,110
TP53	Tumor protein p53	Overexpressed	Overexpressed	111,112
PDK1	Pyruvate dehydrogenase kinase 1	Overexpressed	Underexpressed	113,114
pERK1/2	Extracellular-signal-regulated kinase	Overexpressed	Overexpressed	115,116
PGR/PR	Progesterone receptor	Underexpressed	Underexpressed	90,91
PIK3CA	Phosphatidylinositol 3-kinase	Overexpressed	Underexpressed	117,118
PON1	Paraoxonase/arylesterase 1	Underexpressed	Underexpressed	119,120
PON2	Paraoxonase-2	Overexpressed	Underexpressed	121,122
PTEN	Phosphatase and tensin-homologue as inhibitor	Overexpressed	Underexpressed	117,123
THRA	Thyroid hormone receptor α	Mixed: Overexpressed/Underexpressed	Underexpressed	75,124,125
THRB	Thyroid hormone receptor-β	Underexpressed	Underexpressed	75,126
TNF-alpha	Tumor necrosis factor - alpha	Overexpressed	Overexpressed	127,128
UCP2	uncoupling protein 2	Overexpressed	Underexpressed	75,129
VEGF	Vascular endothelial growth factor	Overexpressed	Overexpressed	130,131

## Conclusion

Breast cancer is the most pervasive cancer in women worldwide and is complex in nature, with multiple subtypes and intricate mechanisms driving its development and progression. There is a well-established association between breast cancer and obesity, with its molecular mechanisms being extensively studied. However, this intricate association is not fully understood. Although the complexities involve hormonal, genetic mechanisms and other genomic factors, this link is still being elucidated. Understanding this relationship is essential for advancing preventive measures, diagnostics and therapeutics that will lessen the burden of cancer care on public health worldwide. Advances in genomics and personalized medicine may offer promising interventions aimed toward obesity-related breast cancer. Moreover, prevention strategies, including lifestyle modifications, including diet, physical activity and weight management, should be considered.

## Data Availability

The authors declare that data supporting the findings of this study are available within the article.
